# Anterior enterocele after cystectomy: case report and review of the literature

**DOI:** 10.1007/s00404-024-07569-0

**Published:** 2024-06-05

**Authors:** Carolin Schröder, Ruben Plöger, Stephanie Knüpfer, Laura Tascón Padrón, Damian J. Ralser, Lucia A. Otten, Eva K. Egger, Alexander Mustea, Dominique Könsgen

**Affiliations:** 1 Department of Gynecology and Gynecological Oncology, University Hospital Bonn, Venusberg Campus 1, 53127 Bonn, Germany; 2 Department of Urology, University Hospital Bonn, Venusberg Campus 1, 53127 Bonn, Germany

**Keywords:** Anterior enterocele, Cystectomy, Pelvic organ prolapse

## Abstract

**Purpose:**

Anterior enterocele is a rare but potentially serious complication after cystectomy with heterogeneous treatment options.

**Methods:**

Here we report on the management of a 71-year-old patient with recurrence of anterior enterocele after cystectomy and provide a systematic review of the literature using the PubMed/MEDLINE database.

**Results:**

The 71-year-old patient with recurrence of anterior enterocele after cystectomy was successfully treated with colpocleisis and anterior colporrhaphy at the Department of Gynecology and Gynecological Oncology, University Hospital Bonn. The use of a synthetic mesh was not needed. At 16-month follow-up postoperatively, the patient was asymptomatic and had no signs of recurrence. *n* = 14 publications including *n* = 39 patients were identified for the systematic review including case reports and reviews. The median duration of developing an anterior enterocele after cystectomy was 9 months (range 3 months to 8 years). Patients had a median age of 71 years (range 44–84). In all cases, a surgical approach was described using a wide variety of surgical procedures. In total, 36% of all patients developed a recurrence with an average time period of 7 months after primary surgery. A rare complication represents a vaginal evisceration with the need of urgent surgery. Furthermore, the occurrence of a fistula is a possible long-term complication.

**Conclusion:**

Anterior enterocele after cystectomy is a rare complication requiring an individual and interdisciplinary treatment.

## What does this study add to the clinical work

**Table:** 

This study is the largest systematic review to describe the heterogenous and complex diagnostic tools and therapeutic approaches of anterior enterocele after cystectomy.	

## Introduction

Pelvic organ prolapse (POP) is a common condition affecting 40 to 60% of parous women, describing the descent of one or more of the pelvic organs. POP can be divided into three compartments: anterior (cystocele, urethrocele, enterocele), middle (uterine), and posterior (rectocele, enterocele) prolapse. Pathophysiologically, POP is a multifactorial disease. Common risk factors compromise age, pregnancies, trauma to the pelvic supporting and connective tissue. Besides, genetic factors that favor connective tissue weakness, lifestyle factors such as body mass index, family history, physical activity, smoking, hormone replacement therapy, etc., are also being discussed as causative factors. POP can also occur after previous operations of pelvic organs. However, the complete etiology is still not fully understood.

Posterior vaginal wall prolapse is commonly referred to as enterocele (when the small intestine and peritoneum are affected) and rectocele (when the rectum is affected). An enterocele can be subdivided into an anterior and a posterior enterocele. While in a posterior enterocele, the small intestine bulges into the pouch of Douglas, in an anterior enterocele, this occurs in the vesicovaginal space. An anterior enterocele is far less common than a posterior and occurs mainly after hysterectomy or cystectomy.

Here, we present a case of a patient with anterior enterocele after cystectomy and provide a systematic review of the literature on the management of anterior enterocele after cystectomy. The aim of the review is to summarize the heterogeneous diagnostic and therapeutic options and to provide a guideline for this rare but potentially serious complication after cystectomy.

## Case report

**Fig. 1:**
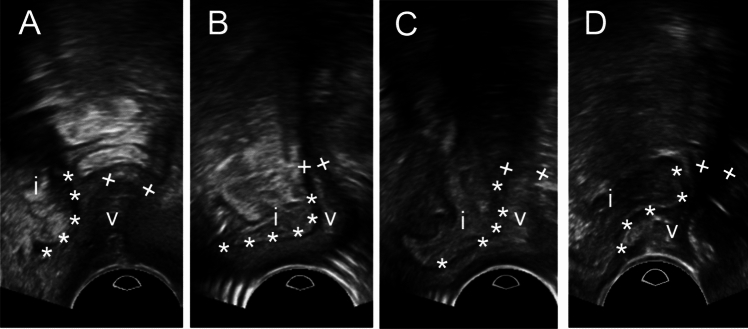
Pelvic floor ultrasound without (**A**) and under Valsalva maneuver (**B–D**) during the patient’s first presentation (**A**, **B**), during the examination after the first operation (**C**), and during the examination after the second operation (**D**). Asterisks mark the border between the intestine (i) and the vaginal stump (v) and plus-signs indicate the end of the vaginal stump. Under Valsalva maneuver, the intestine pushes into the anterior vaginal stump, so that the anterior enterocele can be diagnosed (**B**, **C**)

A 71-year-old female patient presented at our department with a recurrence of an anterior enterocele and prolapse of the vaginal stump (Fig. 1A, B) after anterior colporrhaphy that was performed in an external hospital 1 year before. Previous operations included a hysterectomy and mesh insertion (MESH Prolift™) due to a partial uterine prolapse, cystocele and rectocele, and an anterior exenteration (radical cystectomy) with ileal conduit for papillary urothelial carcinoma of the bladder neck (pT1, p N0 (0/23), L0, V0, R0) 2 years ago. Pre-existing diseases included rheumatoid arthritis, pulmonary fibrosis, arterial hypertension, psoriasis, and hypothyroidism. The obstetric history revealed three complication-free spontaneous vaginal deliveries. After a detailed consultation and explanation of the current findings and possible treatment methods, a colpocleisis according to Conill was performed. In the postoperative course, the patient presented with a second relapse of an anterior enterocele after 2 months (Fig. 1C). Surgical treatment included an adapted colporrhaphy and correction of the enterocele: after dissection of the anterior vaginal wall, the peritoneum—instead of the endopelvic fascia as a result of the previous operations—was dissected, so that adherent regions of the small bowel could be detached from the anterior vaginal wall. A purse-string suture followed by simple interrupted stitches for the double-layer closure of the peritoneum was performed. Finally, the anterior vaginal wall was closed. The follow-up examination after 2 months showed no pathological findings (Fig. 1D). At the latest follow-up 16 months postoperatively, the patient was asymptomatic and had no signs of recurrence.

## Materials and methods

**Fig. 2:**
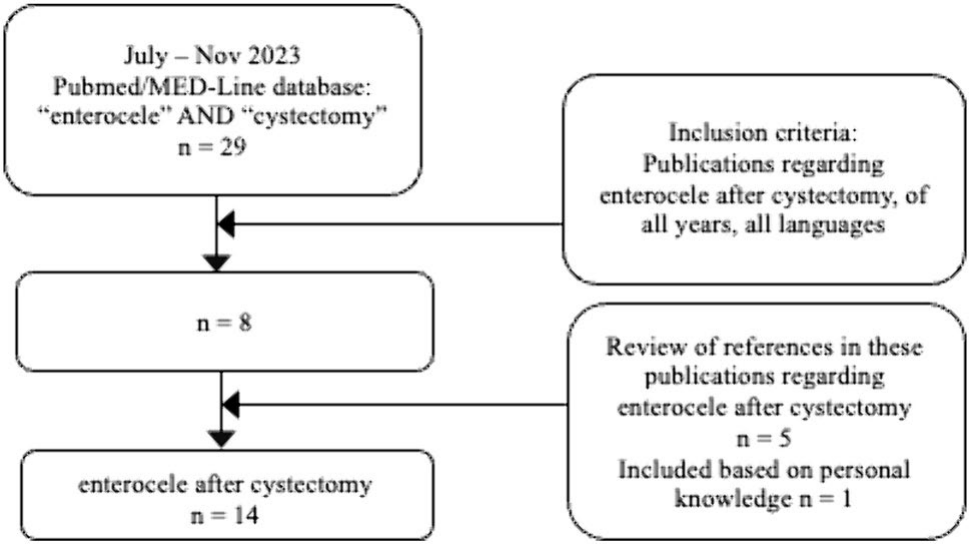
Review of literature

A systematic review was performed using PubMed/MEDLINE database applying the terms “enterocele” and “cystectomy”. Inclusion criteria were publications in any language published up to November 2023 addressing anterior enterocele after cystectomy. *n* = 8 publications met the inclusion criteria. In addition, the references in these eight publications were scanned to find further appropriate publications. *n* = 5 additional publications could be identified. *n* = 1 paper was found and included in this review based on personal knowledge. In total, *n* = 14 publications were included in our review (Fig. 2).

## Results

Our literature search identified *n* = 14 publications with a total of *n* = 39 patients who were diagnosed with an anterior enterocele after cystectomy. The first paper was published in 1998 [1]. The publishing centers were from Israel, Germany, Australia, Japan, and United States of America.

All publications were case reports or case series of which only two publications included a systematic review of the literature [2, 3].

**Fig. 3:**
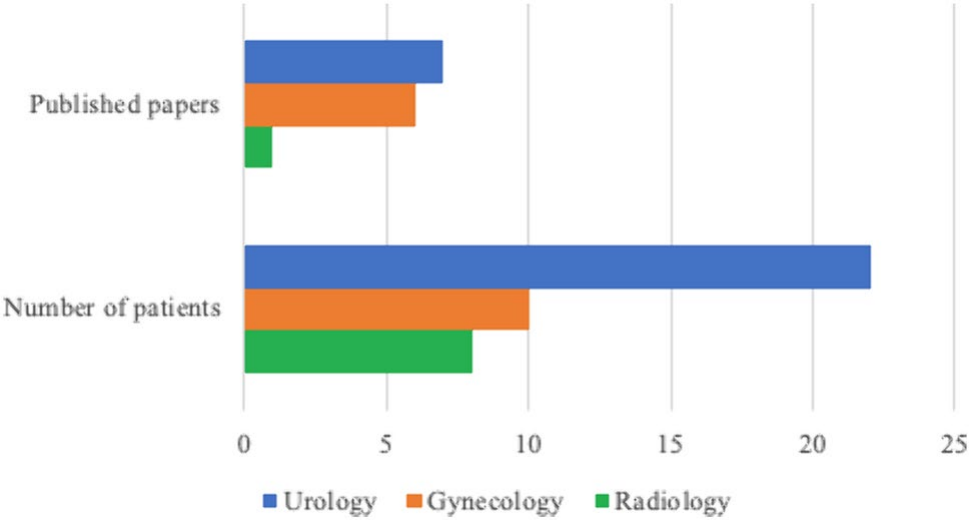
Bar chart of involved departments regarding the published papers and patients

*n* = 7 reports were published by urologists or urogynecologists from a department of urology, corresponding to *n* = 22 patients in total [1–7] (Fig. 3). Only *n* = 6 papers were published by gynecologists, treating *n* = 10 patients [8–13]. One paper, mainly looking at imaging diagnostic tools for anterior enterocele, was published by a radiology department [14]. This distribution corresponds to the heterogeneous treatment methods of anterior enterocele.

The median age at diagnosis was 71 years (range 44 to 84). *n* = 12 patients (31.6%) received a cystectomy including hysterectomy, *n* = 13 patients (33.3%) were operated by cystectomy only. However, these patients had a hysterectomy earlier in life. In *n* = 14 patients (36.8%), there was no information available regarding a previous hysterectomy. The main indication for cystectomy was a muscle invasive bladder cancer (MIBC) in 32 of 39 cases (82.1%). *n* = 5 patients received cystectomy due to interstitial cystitis after failed conservative treatment (13.2%). In *n* = 2 patients, the indication for cystectomy was not reported [4].

### Duration after cystectomy

The median duration between cystectomy and development of an enterocele was 36 weeks. There is, however, a huge range regarding the time of development: the earliest development of an anterior enterocele is described as “shortly after surgery [cystectomy]” [3] and the longest time was 8 years [4].

### POP-Q

Not in all case reports, a pelvic organ prolapse quantification (POP-Q) was stated. However, if available, POP-Q was mainly indicated as stage III or IV, describing a prolapse where the length extends at least > 1 cm beyond hymen [2, 5–10, 14]. In *n* = 10 patients, there was also a prolapse of other compartments such as vaginal vault prolapse or rectocele [1, 3, 4, 11]. Halberthal-Cohen et al. described three cases with POP of all three compartments [11]. A POP-Q stage 0 was reported in two cases by Aziz et al., when looking at the enterocele on MRI [14].

### Symptoms

The main symptom was a vaginal bulge, corresponding to the reported high POP-Q stages [2–4, 12, 13]. Besides, unspecific symptoms such as bleeding or spotting, vaginal discharge, incontinence or ulceration were stated [3–5, 12, 13]. Pain as a symptom was described in only *n* = 4 patients [1, 2, 4, 5]. However, in these three cases, a rare, but potentially serious complication of anterior enterocele, especially after a session of high abdominal pressure was described [2, 5]. Patients complained about acute abdominal pain and vaginal evisceration. Emergency surgery with a prompt correction was indicated. In all cases, a transvaginal approach was used. In two of three cases, a recurrence of enterocele was observed after 4 and 6 months, respectively [2, 5].

### Diagnostic methods

The diagnosis of an anterior enterocele was made clinically by a clinical examination. In addition, in a few cases, MRI (or CT) examination was used to quantify the exact extend of the enterocele and also to exclude a recurrence of the previous cancer [14].

### Treatment

**Fig. 4:**
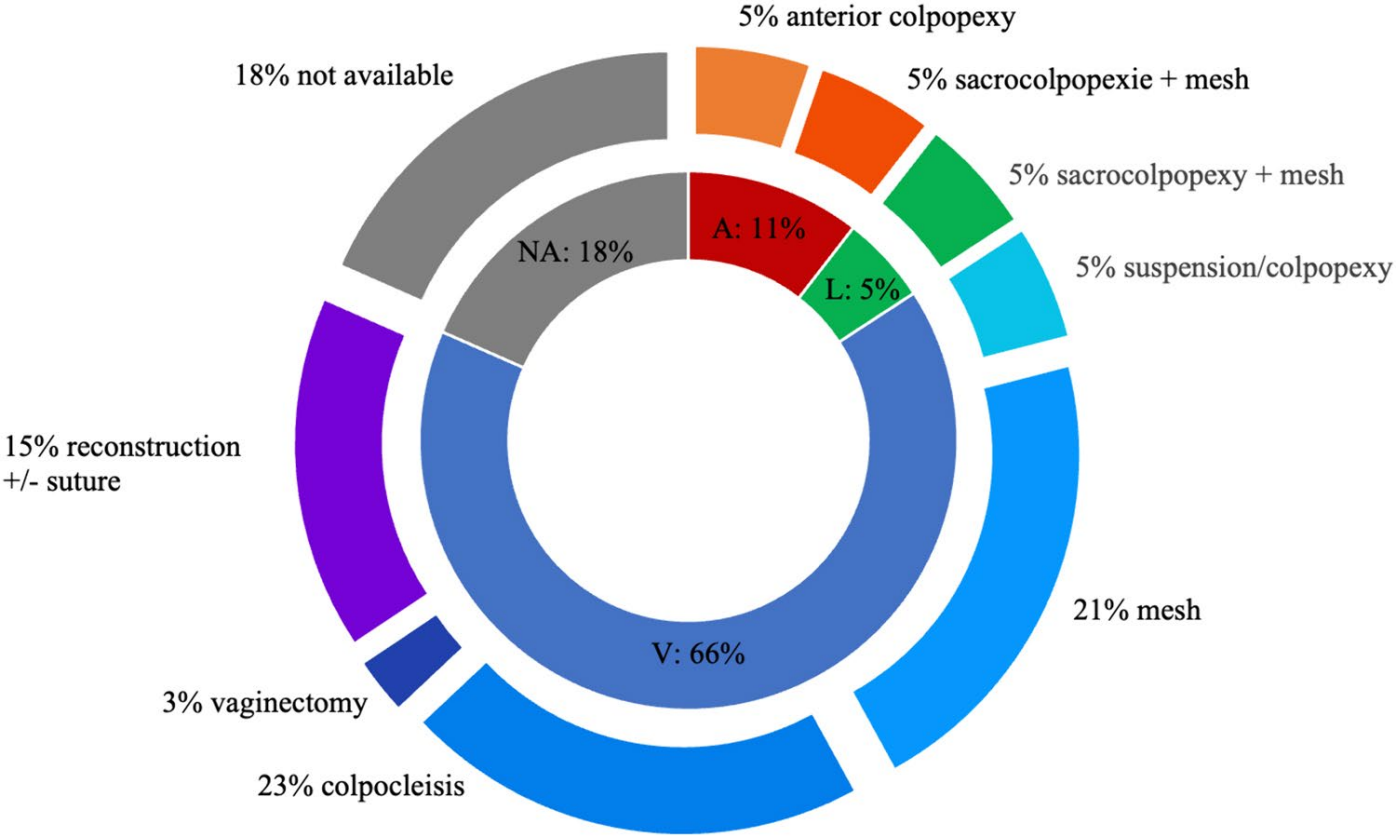
Pie diagram describing the different surgery approaches. *A* abdominal, *L* laparoscopic, *V* vaginal, *NA* not available

The surgical treatment approach in general, regarding existing literature, was very heterogeneous. They can be categorized mainly in three different approaches: abdominal [1, 4], laparoscopic [6], and vaginal [1–3, 5, 7–13] (Fig. 4).

The therapeutic approaches were very different. Vaginal approaches were used more frequently than abdominal or laparoscopic procedures. The main idea of most of the surgeries was a reconstruction using mesh or sutures. In case of older patients, a colpocleisis seemed to be the preferred procedure. Colposuspension or sacrocolpopexy have been done less often; however, when it was performed, a mesh was used in two of three cases.

Pessaries have also been described as a conservative treatment option. However, it was shown that pessary therapy alone was not able to achieve long-term therapeutic success. Pessary therapy was able to bridge the time until surgery as a definitive solution [3].

### Follow-up and recurrence rate

The median follow-up time after any kind of surgery was 10 months with a range from 4 months (in four patients) to a maximum of 60 months. The vast majority of publications only provided information on follow-up in the first 12 months after surgery (22 patients of 39). Only in eight patients, information was provided on the outcome after more than 12 months postoperatively. Interestingly, two of these eight patients had a recurrence after exactly 12 months post-surgery and one of those patients even had two recurrences [2, 5]. Both surgeries included a repair using a biological graft. There was no recurrence after more than 12 months.

There was no correlation between surgical procedure and rate of recurrence: 22% *(n* = 2*)* of the procedures with a recurrence in the first 12 months used a biological mesh [2, 12], 22% *(n* = 2*)* used a synthetic mesh [4, 5]. In 55% of the recurrences during the first year after operation, no mesh was used [2, 3, 5, 11].

When excluding the publication by Aziz et al., omitting information on the recurrence rate, the average recurrence rate is 35.5% (*n* = 11 out of 31). Hence, more than half of the patients did not develop a recurrence after the primary repair of enterocele (64.5%, *n* = 20 of 31). However, patients with a recurrence developed their recurrence in the first 12 months postoperatively, resulting in an average duration of 7 months for the recurrence after primary surgery [2–5, 11, 12].

### Complication

As a long-term complication after correction of an anterior enterocele, the development of a fistula was described twice in the published literature. Fort et al. described a fistula 9 months after surgical repair of the enterocele, which was treated conservatively [12]. Another fistula that was noted only 3 weeks after surgery was reported by Stav et al., and was treated surgically [5].

In one case, a transvaginal partial intestinal resection and anastomosis was necessary due to small intestines fixed along the ulceration of the anterior enterocele [13]. Table 1 summarizes the included papers.

**Table 1:** Summary of included papers

Author year country	Number of cases/study type	Specialty of 1st author	Age at diagnosis (years)	Previous operation (indication)	Duration until enterocele	POP-Q at diagnosis	Symptoms of patient	Treatment	Recurrence (treatment)
Anderson (1) 1998 USA	3 case series	Uro	(1) 71 (2) 56 (3) 61	(1–3) Cystectomy (interst. cystitis), prior hysterectomy	(1) 8 months (2) 14 months (3) 16 months	(1) AE (2) AE (3) AE + vaginal vault prolapse	(1) Mass protruding at introitus (2) Heaviness in perineal area (3) Pain and mass in perineal area	(1) Transabdominal anterior colpopexy, Moschcowitz procedure (2) Transabdominal anterior colpopexy (3) Transvaginal sacrospinous colpopexy	None at (1) 12 months (2) 19 months (3) 33 months
Stav (5) 2008 Australia	5 case series	Uro	(1) 70 (2) 71 (3) 69 (4) 44 (5) 65	(1, 2, 4, 5) Radical cystectomy (MIBC) (3) Radical cystectomy (MIBC), prior hysterectomy	(1) 16 months (2) 18 months (3) 10 months (4) 2 months (5) 7 months	(1) Stage III AE (2) Stage IV AE (3) Stage IV AE (4) Stage IV AE (5) Complete intestinal evisceration	(1) NA (2) Superficial vaginal ulceration (3) Vaginal ulceration (4) NA (5) Acute abdominal pain	(1) Transvaginal, biological mesh (2) Colpocleisis (3) Anterior vaginectomy (4) Iliococcygeal suspension with synthetic mesh reinforcement (5) Colpocleisis	(1) 12 months stage III recurrence (transobturator mesh), no recurrence after 2 years (2) None at 10 months follow-up (3) None at 5 months follow-up (4) 3 weeks: fistula (surgery), no recurrence after 10 months (5) Recurrence at 6 months follow-up (surgery)
Graefe (8) 2012 Germany	2 case series	Gyn	(1) 67 (2) 76	(1 + 2) Cystectomy (MIBC)	(1) 8 months (2) 12 months	(1 + 2) Stage IV	NA	(1 + 2) Interposition of an Elevate® Anterior	None at (1) 16 months (2) 4 months
Abe (6) 2019 Japan	2 case series	Uro	(1) 78 (2) 78	(1 + 2) Cystectomy (MIBC)	NA	(1 + 2) Stage IV	NA	(1 + 2) Laparoscopic sacrocolpopexy (mesh)	None at (1) 18 months (2) 4 months
Lin (2) 2019 USA	5 case series and review	Uro	(1) 55 (2) 68 (3) 73 (4) 73 (5) 79	(1) Robotic cystectomy (MIBC), years earlier hysterectomy (benign) (2) Robotic cystectomy (MIBC), prior hysterectomy (benign) (3–5) Robotic cystectomy + hysterectomy (MIBC)	(1) 16 weeks (2) 56 weeks (3) 14 weeks (4) 120 weeks (5) 11 weeks	(1) Stage IV (3 + 4) Complete intestinal evisceration (2 + 5) NA	(1) Recurrence after colpocleisis externally (2) Vaginal bulge (3) Vaginal bulge, acute bowel evisceration through vaginal wall defect after coughing episode (4) Vaginal bulge, patient eviscerated vaginally (5) Vaginal discharge + bulge	(1) Excision of enterocele sac, biological graft, perineorrhaphy (2) Transvaginal reconstruction (3) Transvaginal placement of several rows of suture on posterior aspect of pubic bone with absorbable suture (4) Transvaginal reduction of prolapsed bowel, repair of the vaginal cuff dehiscence, colpocleisis, levatorplasty, and perineorrhaphy (5) Repair of enterocele, colpocleisis, perineorrhaphy	(1) After 12 months recurrence (3rd repair transvaginally Moschcowitz operation, after 6 months recurrence (complete perineorrhaphy)), no recurrence at 5-year follow-up (2) After 6 months stage II recurrence (partial vaginectomy), after 6 weeks recurrence (transvaginal biological graft, approximating the posterior vaginal wall to the pubic symphysis perineorrhaphy) (3) None at 11 months follow-up (4) Recurrence 4 months later (colpocleisis with biologic augment, extended perineorraphy), 3 weeks afterward no recurrence (5) None at 8 months follow-up
Zimmern (4) 2019 USA	2 case series	Uro	(1) 71 (2) 72	(1 + 2) Cystectomy, prior hysterectomy	(1) 8 years (2) 1.5 years	(1) Stage III vaginal vault prolapse (2) Stage IV AE, vaginal vault prolapse	(1) Pelvic pressure/pain, vaginal bulge, fecal incontinence (2) Vaginal bulge, vaginal spotting	(1 + 2) Abdominal sacrocolpopexy + mesh, omental flap wrap	(1) None at 45 months follow-up (2) At 11 months: small AE recurrence (transvaginal anterior colporrhaphy), 12 months afterward asymptomatic
Cruz (3) 2020 USA	4 case series and review	Uro	(1) 77 (2) 72 (3) 69 (4) 77	(1) Cystectomy (MIBC) (2) Cystectomy (MIBC), prior hysterectomy (3) Cystectomy (MIBC) (4) Cystectomy (MIBC), prior hysterectomy (fibroids)	(1) 11 months (2) 7 months (3) Several years (4) NA (shortly after surgery)	(1) Stage IV AE, stage II rectocele (2) Stage IV AE, stage II rectocele (3) Stage II AE, stage III rectocele (4) Stage IV AE, stage III rectocele	(1) Bladder pressure, vaginal bulge (2) Vaginal bulge, bleeding, discharge (3 + 4) Vaginal bulge	(1 + 4) Transvaginal enterocele repair, vaginal vault suspension, rectocele repair, perineoplasty (2) Colpocleisis, perineoplasty (3) Colpocleisis, perineoplasty	(1) None at 8 months follow-up (2) At 10 months prolonged ileus + recurrence (pessary, vaginal vault suspension) (3) None at 22 months follow-up (4) None at 6 months follow-up
Fort (12) 2020 USA	2 case series	Gyn	(1) 78 (2) 46	(1) Robotic cystectomy + hysterectomy (MIBC) (2) Cystectomy (MIBC), prior hysterectomy	(1) 4 months (2) 3 weeks	(1) AE (2) Small bowel evisceration into vaginal canal	(1) Vaginal bulge (2) Vaginal fulness, fluid drainage	(1 + 2) Transvaginal reconstruction of anterior vaginal wall using xenograft	(1) At 9 months follow-up: colovaginal fistula (conservatively), at 1 year follow-up: asymptomatic (2) None at 1-year follow-up
Halberthal- Cohen (11) 2019 Israel	3 case series	Gyn	(1–3) 60–80	(1–3) Cystectomy (MIBC), two patients additionally hysterectomy	NA	(1–3) POP of all three compartments	NA	(1 + 2) Colpocleisis (3) Latter procedure (vaginal closure)	(1 + 2) Recurrence after 2–4 months (side-to-side closure of the vagina), after another 4 months, no recurrence (3) None at 4-month follow-up
Okada (9) 2020 Japan	1 case report	Gyn	(1) 78	(1) Cystectomy (MIBC) + hysterectomy	(1) 3 months	(1) Stage III AE	(1) Vaginal bulge	(1) Transvaginal reconstruction of anterior vaginal wall using mesh, Martius labial fat pad flap	(1) None at 6-month follow-up
Shaker (10) 2020 Australia	1 case report	Gyn	(1) 75	(1) Cystectomy (MIBC), prior hysterectomy	(1) 10 months	(1) Stage III AE	(1) Vaginal bulge	(1) Transvaginal reconstruction of vaginal wall using suture	(1) None at 4-month follow-up
Sandru (13) 2020 Germany	1 case report	Gyn	(1) 84	(1) Cystectomy (BC), prior hysterectomy	(1) NA	(1) Stage IV	(1) Vaginal bulge, bleeding, ulceration	(1) Colpectomy, colpocleisis, partial intestinal resection with anastomosis	NA
Aziz (14) 2021 USA	7 case series	Radio	(1) 81 (2) 70 (3) 72 (4) 59 (5) 71 (6) 66 (7) 62	(1 + 5) Cystectomy (cystitis) (2–4, 6 + 7) Cystectomy (MIBC) + hysterectomy	(1) 72 months (2) 108 months (3) 12 months (4) 2 months (5) 5 months (6) 9 months (7) 8 months	(1) Stage II AE (2) Stage III AE (3) Stage III AE (4) Stage II AE (5) Stage II AE (6) Stage 0 (7) Stage 0	NA	NA	NA
Kuwata (7) 2022 Japan	1 case report	Uro	(1) 84	(1) Robotic cystectomy (MIBC)	(1) 4 months	(1) Stage IV AE	NA	(1) Transvaginal repair using a mesh	(1) None at 1 year follow-up

*NA* not available, *AE* anterior enterocele, *MIBC* muscle invasive bladder cancer, *BC* bladder cancer

## Discussion

Treatment of an anterior enterocele after cystectomy is challenging. The exact incidence remains unclear. Even though only a few case reports have been published, an additional number of unreported cases can be assumed, particularly considering the fact that a generally benign disorder is likely to be underrepresented in patients with an underlying malignant disease. Further, an anterior enterocele might be underdiagnosed in patients with disease progression or short survival due to the cancer diagnosis.

POP is a multifactorial disease with different common risk factors. Women, in particular, are at higher risk for developing POP after radical cystectomy as a tissue weakening operation followed by chemotherapy.

In Germany, approximately 18,270 people developed invasive bladder cancer in 2018, a quarter of whom were women [15]. Although the ratio of male to female is 4:1, women tend to have more aggressive disease requiring radical management [16]. In non-metastatic muscle invasive bladder cancer, radical cystectomy (RC) with pelvic lymph node dissection is the gold standard therapy.

In women, the proximity of the vagina and uterus to the bladder carries the risk of adjacent spread of cancer. Therefore, RC is often performed as part of an anterior exenteration with concurrent removal of the bladder, uterus, and anterior wall of the vagina [17].

The postoperative complication of vaginal prolapse after RC has been rarely systematically investigated to date. This might be due to the hindered follow‐up on the basis of varied study designs. Although case series of individual complications have been reported, the actual incidence remains unknown [18, 19]. As the focus has been on oncological cure and therapy, the diagnosis and treatment of vaginal prolapse may be delayed or even forgotten [5].

Recently, patient-reported outcome measures (PROMS) after RC regarding vaginal prolapse, as well as structured vaginal examination, have been systematically evaluated in a study by Wenk et al. using three validated questionnaires (International Consultation on Incontinence Questionnaire Vaginal Symptoms [ICIQ-VS] Part A, Pelvic Organ Prolapse/Urinary Incontinence Sexual Questionnaire IUGA revised [PISQ], European Organization for Research and Treatment of Cancer Quality of Life Questionnaire [EORTC] C30/BLM30) [20]. Wenk and colleagues detected in their study group that the type of urinary diversion, POP-Q stages, and tumor stages did not show significant differences regarding sexual function, QoL, and prolapse complaints in women after RC, whereas a vagina-sparing approach showed significant differences only in two subscales without clinical relevance. This result has also been demonstrated by a study from Lipetskaia et al [21]. The study quoted the absence of correlation between radical cystectomy for uroepithelial carcinoma with an increased risk of patient-reported symptoms of pelvic organ prolapse. Unfortunately, most of the published studies are either case reports or small sample size reviewed retrospective. Hence, a substantial statement is missing and a prospective evaluation using standardized instruments is needed.

As to date, there is no preventing procedure for anterior enterocele after cystectomy. Patients with risk factors should be informed preoperatively about potential risks after cystectomy. Potential risk factors include previous hysterectomy, increasing age, and previous prolapse symptoms. Interestingly, Okada et al. performed a pathological examination of the anterior vaginal wall after enterocele, resulting in inflammatory granulation tissue and adhesions [9]. The idea, that targeted pelvic floor exercises with additional local estrogenization and pessary therapy after cystectomy might prevent the occurrence of an anterior enterocele, is purely hypothetical. To prevent an inflammatory response, administration of antibiotics vaginally or systemically is also being discussed. Unfortunately, there is no conclusive data available on this topic.

It is suggested by Okada et al., that an anterior enterocele can be subdivided into two types: one that still contains the full thickness of the vaginal wall and the other types that does not [9]. This might underline the opinion of Stav et al., who suspect that pessary therapy could provoke the risk of erosion or evisceration of the already thinned vaginal wall [5]. Finally, it is evident that only a few papers include pessary therapy as a conservative therapy option, which might as well be due to publication bias.

That a surgical approach after cystectomy, however, might be difficult can be explained by the anatomic changes and the new locations of the ureters after cystectomy. That is why a laparoscopic or abdominal insertion of a mesh might be more difficult. Adhesions might additionally be another reason [4]. Re-peritonealization to cover a mesh can be challenging after cystectomy due to the missing tissue. Zimmern et al. describe an omental flap wrap as an alternative approach for abdominal sacrocolpopexy [4]. However, a transvaginal reposition of the anterior enterocele is also challenging due to the mostly missing endopelvic fascia after radical cystectomy. Here as well, a biological tissue flap was used by Okada et al.: fat tissue from one labia majora was used to avoid direct contact between the peritoneum and the mesh (“Martius labial fat pad”) [9].

Considering the advantages and disadvantages of homologous or synthetic mesh repair, there seems to be no preferred procedure that lowers the recurrence rate. Overall, it seems to be rather important to choose an individual regime, ideally within an interdisciplinary setting.

Prior primary repair, patients should be informed about the high risk of recurrence. That is why it seems reasonable to choose a homologous mesh repair for the primary operation first. The use of a synthetic mesh is still an option for the relapse setting. As we could show in our case report, a colpocleisis followed by an adapted colporrhaphy and correction of the enterocele after recurrence seemed to be the right decision for our patient. This was discussed with the patient during detailed consultations and explanations of the current findings including all possible treatment methods.

## Conclusion

AE is a rare, but potentially serious complication after cystectomy that should be informed to patient’s prior surgery. Even though anterior enterocele represents a benign complication, it can affect the quality of patient’s life and should, therefore, be approached seriously. Due to the rare cases and different treatment approaches, there is no treatment path that fits for every patient. An individual regime including the patient’s history and preference should be pursued, ideally in an interdisciplinary setting. Therefore, the development of a SOP (standard operating procedure) as well as a prospective registry study due to the limited data available should be aimed.

Patients should be informed about the high risk of recurrence. This review emphasizes the high need of long-term studies and follow-up after surgery for anterior enterocele.

## Data Availability

Dataset and materials used in this study are available from the corresponding author upon reasonable request.
